# Differences and Interactions in Placental Manganese and Iron Transfer across an In Vitro Model of Human Villous Trophoblasts

**DOI:** 10.3390/ijms23063296

**Published:** 2022-03-18

**Authors:** Vivien Michaelis, Leonie Aengenheister, Max Tuchtenhagen, Jörg Rinklebe, Franziska Ebert, Tanja Schwerdtle, Tina Buerki-Thurnherr, Julia Bornhorst

**Affiliations:** 1 Food Chemistry, Faculty of Mathematics and Natural Sciences, University of Wuppertal, Gaußstraße 20, 42119 Wuppertal, Germany; vivien.michaelis@uni-wuppertal.de; 2 Swiss Federal Laboratories for Materials Science and Technology, Empa, Particles-Biology Interactions, Lerchenfeldstraße 5, 9014 St. Gallen, Switzerland; leonie.aengenheister@lih.lu (L.A.); tina.buerki@empa.ch (T.B.-T.); 3 Luxembourg Institute of Health, Rue Thomas Edison 1 A–B, 1445 Strassen, Luxembourg; 4 TraceAge-DFG Research Unit on Interactions of Essential Trace Elements in Healthy and Diseased Elderly (FOR 2558), Berlin-Potsdam-Jena-Wuppertal, 14558 Nuthetal, Germany; tuchtenhagen@uni-potsdam.de (M.T.); tanja.schwerdtle@uni-potsdam.de (T.S.); 5 Department of Food Chemistry, Institute of Nutritional Science, University of Potsdam, Arthur-Scheunert-Allee 114–116, 14558 Nuthetal, Germany; fraebert@uni-potsdam.de; 6 Laboratory of Soil, and Groundwater-Management, School of Architecture and Civil Engineering, Institute of Foundation Engineering, Water- and Waste-Management, University of Wuppertal, Pauluskirchstraße 7, 42285 Wuppertal, Germany; rinklebe@uni-wuppertal.de; 7 German Federal Institute for Risk Assessment (BfR), Max-Dohrn-Straße 8-10, 10589 Berlin, Germany

**Keywords:** manganese, iron, placental transfer, TE interactions, BeWo b30 trophoblasts

## Abstract

Manganese (Mn) as well as iron (Fe) are essential trace elements (TE) important for the maintenance of physiological functions including fetal development. However, in the case of Mn, evidence suggests that excess levels of intrauterine Mn are associated with adverse pregnancy outcomes. Although Mn is known to cross the placenta, the fundamentals of Mn transfer kinetics and mechanisms are largely unknown. Moreover, exposure to combinations of TEs should be considered in mechanistic transfer studies, in particular for TEs expected to share similar transfer pathways. Here, we performed a mechanistic in vitro study on the placental transfer of Mn across a BeWo b30 trophoblast layer. Our data revealed distinct differences in the placental transfer of Mn and Fe. While placental permeability to Fe showed a clear inverse dose-dependency, Mn transfer was largely independent of the applied doses. Concurrent exposure of Mn and Fe revealed transfer interactions of Fe and Mn, indicating that they share common transfer mechanisms. In general, mRNA and protein expression of discussed transporters like DMT1, TfR, or FPN were only marginally altered in BeWo cells despite the different exposure scenarios highlighting that Mn transfer across the trophoblast layer likely involves a combination of active and passive transport processes.

## 1. Introduction

Although trace elements (TE) like manganese (Mn) and iron (Fe) are essential to sustain physiological processes, many studies have shown that excessive metal uptake could lead to health issues such as neurodegenerative diseases with metal-induced oxidative stress as a potential underlying pathway [1,2]. Mn and Fe may cause oxidative stress directly induced by Fenton and Fenton-like reactions or indirectly by inhibition of mitochondrial respiratory chain complexes [3,4]. This adverse outcome of excessive Mn and Fe exposure is presently established for adults, but may be of particular concern for sensitive populations like expecting mothers and their unborn children. In early stages of neurodevelopment, the neuronal network is highly susceptible to oxidative damage due to a not fully developed antioxidant system and a high oxygen consumption rate [5]. Therefore, an efficient TE homeostasis has to be maintained to avoid accumulation of metal species or reactive oxygen species (ROS) and ensure optimal fetal (neuro)development [6]. Maternal-fetal TE transfer is tightly regulated by the placenta, a highly species-specific organ performing a variety of essential functions including the exchange of nutrients in order to sustain fetal development. Among the many cell types forming the placental barrier, the syncytiotrophoblast facing the maternal blood stream is the key barrier layer which performs most pregnancy-relevant functions and expresses a wealth of enzymes and transporters. Early in pregnancy, cytotrophoblast precursor cells expand and differentiate from a bilayer to a thin multinuclear syncytiotrophoblast (2–4 µm thickness), which leads to an increased placental permeability in later stages of pregnancy [7,8]. While the transfer of Fe across placental barrier building cells has already been addressed in numerous studies (reviewed in [9,10]), little is known about the transfer of Mn. 

Mn is involved in several processes of fetal development like growth, bone formation, immune function, or neurodevelopment [11,12]. Since Mn appears ubiquitously in nutrition (e.g., nuts, grains, rice, and tea) but also in the environment through anthropogenic pollution of air or drinking water, Mn deficiency has not been observed in humans so far [13,14]. However, excess levels of Mn in utero (measured as maternal blood and/or cord blood concentrations) have been associated with adverse pregnancy outcomes such as a higher risk for intrauterine growth restriction or neural tube defects, lower birth weight, or an altered neurodevelopment in terms of psychomotor and mental skills [15,16,17,18]. The underlying toxicity mechanisms are still unknown [19,20]. For adults including pregnant or lactating women, the European Food Safety Authority (EFSA) proposed an adequate Mn intake (AI) of 3 mg/ day (2013), which relies on extrapolated data from the Institute of Medicine, Panel on Micronutrients on weight gain during pregnancy (2001) [14,21]. Nonetheless, Mn requirement in pregnancy is still under discussion. 

To identify possible placental TE transfer mechanisms, Duck et al. reviewed Fe transfer across different physiological barriers including the placenta [22]. Different from Fe transfer in the gut, placental Fe transfer is described as a unidirectional (from mother to child only) transferrin (Tf) mediated endocytosis including active transport mechanisms. In more detail, Tf-bound trivalent Fe(III) (Tf-Fe) is transported to the endosome where it is pH-dependently released and subsequently transported into the cytosol through the divalent metal transporter 1 (DMT1). Once in the cytosol, it can be stored in the storage proteins ferritin light and heavy chain (FTL, FTH) or exported to the fetal side through ferroportin (FPN) [22]. The limited data available on placental Mn transfer suggest that Mn is transported actively since the Mn amount was significantly higher in umbilical cord blood than in maternal serum [23]. In addition, the Mn transfer rate across a perfused human placental lobule was reduced compared to the passive diffusion marker antipyrine [15]. Nevertheless, further mechanistic studies on placental Mn transfer and safety, including transfer kinetics as well as underlying translocation and toxicity mechanisms are urgently needed to understand micronutrient requirements in pregnancy [24]. 

Therefore, we investigated placental Mn transfer and cytotoxicity at the villous trophoblast barrier using an in vitro BeWo b30 transfer model. In particular, we focused on elucidating Mn transfer kinetics and underlying translocation pathways like the involvement of relevant transporters (DMT1, TfR, and FPN) and regulatory proteins (metallothioneins (MT), FTH, FTL). As previously described for other tissues, Mn and Fe might share the same transport mechanisms because they both occur in two physiological relevant species ((+II) and (+III)) [25,26]. Hence, we included Fe in our study for comparison. This study helps to fill the knowledge gaps in the transfer of single TEs but also emphasizes the importance of TE interactions in order to maintain a balanced TE homeostasis. 

## 2. Results

### 2.1. Mn and Fe Cytotoxicity in Confluent BeWo b30 Cells

The cytotoxicity of MnCl_2_ and FeCl_2_ was assessed in confluent BeWo b30 cells to determine non-toxic concentration ranges for transfer experiments. Indirect determination of the cell number via Hoechst assay after 24 h showed no effect for concentrations up to 1000 µM of either MnCl_2_ or FeCl_2_ (Figure S1). Therefore, concentrations between 100–1000 µM MnCl_2_ or 10–500 µM FeCl_2_ were applied for single element transfer and 100 µM MnCl_2_ and 10–100 µM FeCl_2_ for combination studies.

### 2.2. Mn and Fe Transfer across the BeWo b30 Cell Layer

Growing BeWo b30 cells to a confluent and polarized layer on microporous inserts allows the investigation of Mn and Fe transfer across two chambers where the apical chamber refers to the maternal side and the basolateral chamber to the fetal side of the placental barrier.

BeWo barrier integrity and tightness was verified by measuring the transepithelial electrical resistance (TEER), sodium fluorescein exclusion, and immunocytochemical staining of adherence junction (γ-catenin) and microtubule proteins (tubulin) as described in Aengenheister et al. [27]. TEER reached values of 300 ± 30 Ω*cm^2^ and capacitance values of 2.8 ± 1.1 µF/cm^2^ after 3 days of cultivation on inserts and remained at this level throughout the duration of the transfer experiment. Transwells^®^ with TEER values exceeding 330 Ω*cm^2^ were not used for transfer experiments since BeWo b30 cells do not undergo contact inhibition of growth and formed a multilayer already within the 24 h of TE exposure, which led to a compromised TE transfer (data not shown) [28].

**Figure 1:**
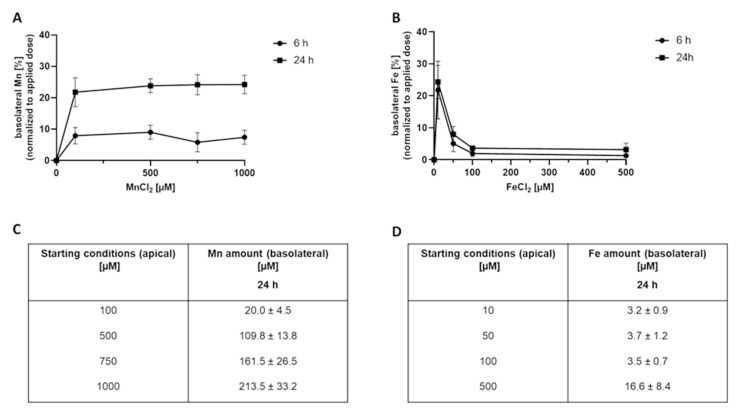
Basolateral Mn and Fe amounts 6 h and 24 h after MnCl_2_ or FeCl_2_ treatment of confluent BeWo b30 cells. Data are presented as mean ± SD of three independent experiments with two replicates each. Figures (**A**,**B**) show the basolateral Mn and Fe amount [%] normalized to the applied dose after 6 h and 24 h. Tables (**C**,**D**) show the respective basolateral amount [µM] exemplarily after 24 h.

Applying Mn to the apical side and quantifying the basolateral Mn amount revealed a time- and concentration-dependent Mn transfer across the BeWo b30 cell layer (Figure 1A,C). Interestingly, normalization to the applied dose (Figure 1A) showed that Mn transfer amounts are comparable in all applied doses in the range of 8 ± 3% after 6 h and 21 ± 5% after 24 h. However, applying Fe to the apical side, the basolateral Fe amount normalized to the applied dose (Figure 1B) decreased from 24 ± 5% (10 µM FeCl_2_, 24 h) to 4 ± 2% (500 µM FeCl_2_, 24 h) with increasing FeCl_2_ incubation concentration. Concentrations higher than 100 µM lead to a plateau with only 4 ± 1% of applied FeCl_2_, indicating a different transfer mechanism compared to Mn.

**Figure 2:**
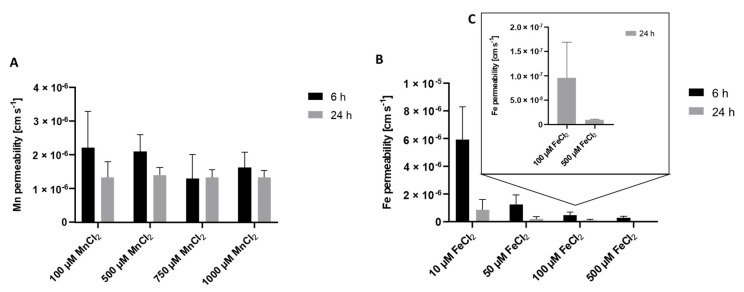
Mn and Fe permeability coefficients across the confluent BeWo b30 cell layer. Permeability was determined in regards to apical Mn or Fe treatment after 6 h and 24 h. Shown is the mean + SD of at least three replicates each. (**A**) Mn permeability, (**B**) Fe permeability, (**C**) enlarged section of permeability coefficients of 100 µM FeCl_2_ and 500 µM FeCl_2_ after 24 h.

To get a better understanding of the underlying transfer kinetics, the permeability of Mn as well as Fe was determined. The permeability coefficient allows comparing Mn and Fe transfer to other marker substances such as antipyrine as carried out by Aengenheister et al. [27] or other in vitro and ex vivo models [29]. Incubating 100 µM MnCl_2_ for 24 h on the apical side resulted in a total crossover of 21.4 ± 4.6% to the basolateral side with a defined permeability of 1.2 × 10^−6^ ± 3.8 × 10^−7^ cm/s. Since percental Mn crossover was concentration-independent (Figure 1A), similar permeability coefficients were obtained for all applied Mn concentrations (Figure 2A). Incubating 100 µM FeCl_2_ resulted in a crossover to the basolateral side of only 3.7 ± 0.8% and a permeability of 9.5 × 10^−8^ ± 7.4 × 10^−8^ cm/s (Figure 2C), which is less compared to Mn transfer rates. In comparison, FeCl_2_ permeability was higher for the incubation of lower concentrations like 50 µM FeCl_2_ (1.3 × 10^−6^ ± 6.7 × 10^−7^) and lower at higher FeCl_2_ concentrations like 500 µM FeCl_2_ (3.0 × 10^−7^ ± 1.1 × 10^−7^) (Figure 2B).

### 2.3. Mn and Fe Transfer Interactions

**Figure 3:**
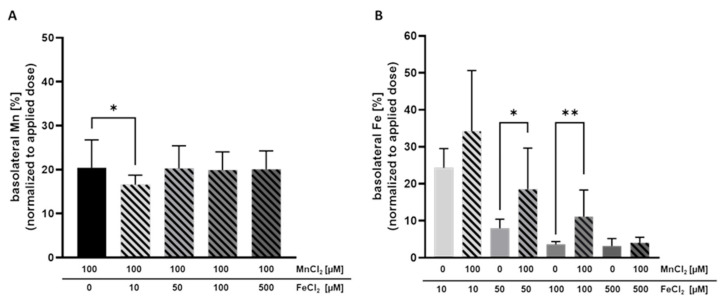
Basolateral Mn and Fe amount after concurrent treatment after 24 h. Shown is the mean + SD of at least three independent experiments with two replicates each. (**A**) Basolateral Mn amount [%] normalized to the applied dose. (**B**) Basolateral Fe amount [%] normalized to the applied dose. Statistical analysis is based on an unpaired *t* test with Welch’s correction compared to single TE treatment. Statistical analysis is indicated as followed: * *p* < 0.05, ** *p* < 0.01 compared to single TE treatment.

To further characterize Mn transfer in terms of transporter involvement and a potential competition with Fe, Mn and Fe were concurrently incubated on the apical side of the BeWo b30 layer for 24 h. Therefore, the concentration of MnCl_2_ was set to 100 µM while various FeCl_2_ concentrations ranging from 10 µM–500 µM were applied. Simultaneous incubation with 100 µM MnCl_2_ and 10 µM FeCl_2_ for 24 h significantly decreased Mn transfer. While without Fe, the basolateral Mn amount was 21.4 ± 4.6% normalized to the applied dose; only 14.2 ± 1.9% were transferred to the basolateral side in combination with 10 µM FeCl_2_ (Figure 3A). However, co-exposing BeWo b30 cells with either 50 µM FeCl_2_ or 100 µM FeCl_2_ and 100 µM MnCl_2_ showed no impact on the transferred Mn amounts. 

Mn transfer and Fe transfer were both altered by combined Mn and Fe exposure. Incubating 50 µM FeCl_2_ or 100 µM FeCl_2_ with 100 µM MnCl_2_ resulted in a significantly increased Fe transfer of 18.6 ± 11.1% vs. 8.0 ± 2.4% (Fe only) and 11.1 ± 7.2% vs. 3.7 ± 0.8% (Fe only), respectively (Figure 3B). Since Fe exhibited an impact on Mn transfer and also Mn affected the Fe transfer, it could be assumed that they share similar transport systems in trophoblast cells. 

### 2.4. Cellular Amount of Mn and Fe

**Figure 4:**
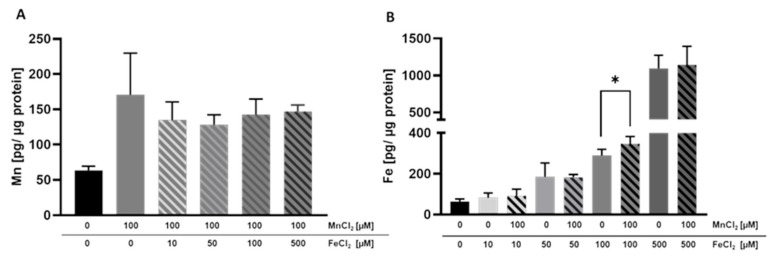
Mn and Fe bioavailability in BeWo b30 cells grown on inserts. BeWo b30 cells were incubated with MnCl_2_ and/or FeCl_2_ for 48 h, respectively. Total Mn or Fe amount was determined analytically using ICP-OES or ICP-MS/MS. (**A**) Mn amount [pg Mn/ µg protein]. (**B**) Fe amount [pg Fe/ µg protein]. Shown is the mean + SD of three independent experiments with two replicates each. Statistical analysis which is based on an unpaired *t* test is indicated as followed: *: compared to single TE treatment.

Alongside the transferred amounts of Mn and Fe, we quantified the cellular amount of these TEs in the BeWo b30 cells. Single exposure to MnCl_2_ or FeCl_2_ resulted in an increased cellular content, indicating that Mn and Fe are taken up by the cells (Figure 4A,B). Cellular Mn levels were not considerably altered by combinational incubation with FeCl_2_. However, the trend of a slight decrease in the cellular Mn content was observed at all FeCl_2_ concentrations, which may be a first indication that Mn bioavailability could be compromised in the presence of high Fe levels (Figure 4A). In contrast, Fe uptake was significantly increased in cells treated with 100 µM FeCl_2_ + 100 µM MnCl_2_ compared to single Fe treatment (Figure 4B), but in the other combinations, the cellular concentrations were indistinguishable from single Fe exposure.

### 2.5. mRNA and Protein Expression of Mn and Fe Associated Genes and Related Proteins

**Figure 5:**
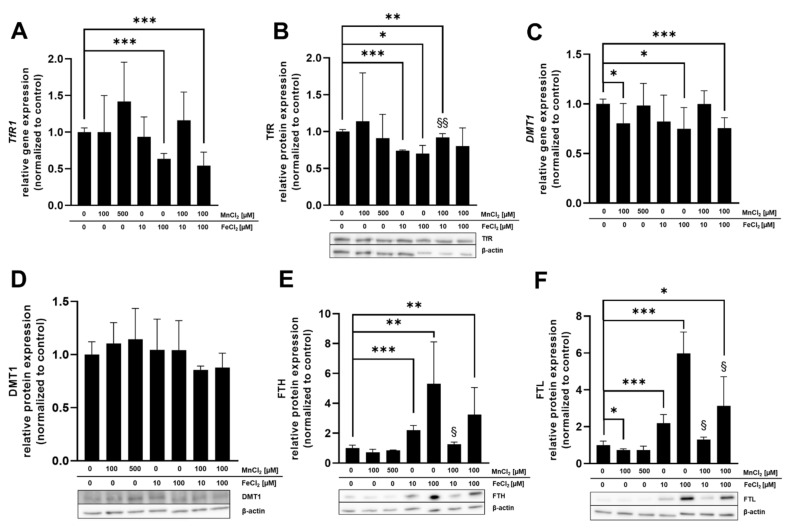

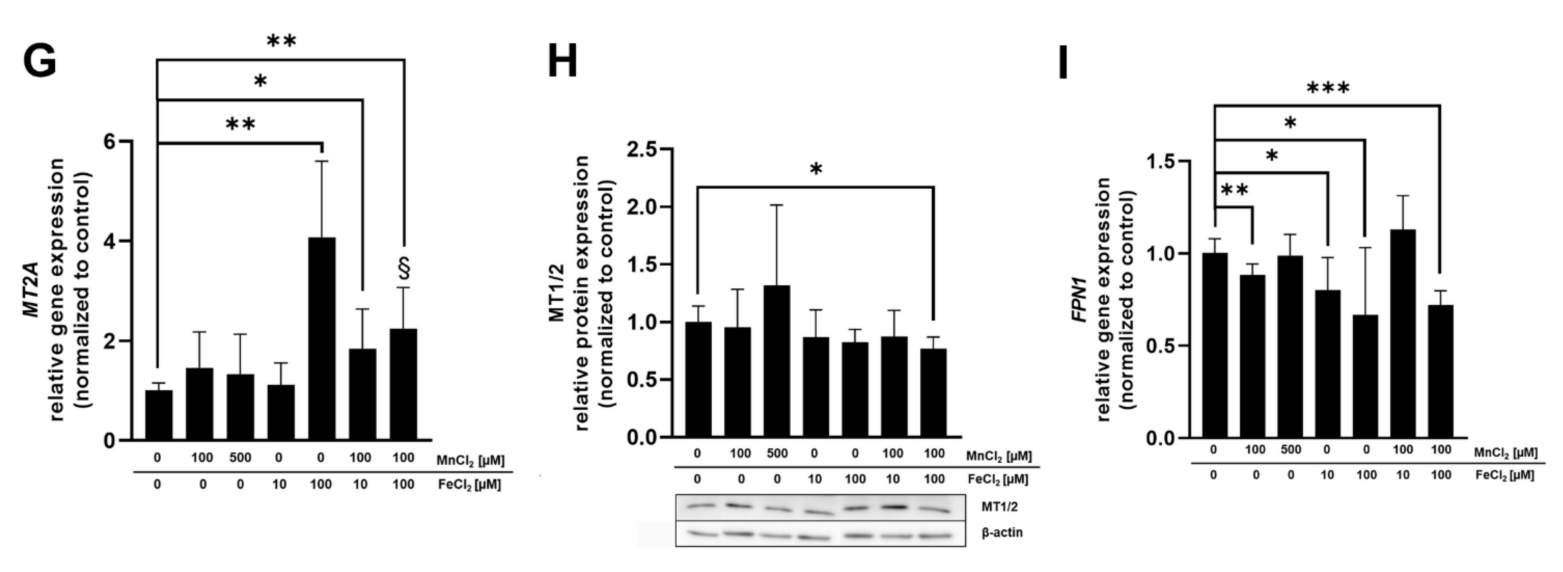
Relative mRNA and protein levels of Mn and Fe transport- and storage-associated genes and their respective proteins. (**A**) *TfR1* relative gene expression. (**B**) TfR relative protein expression. (**C**) *DMT1* relative gene expression. (**D**) DMT1 relative protein expression. (**E**) FTH relative protein expression. (**F**) FTL relative protein expression. (**G**) *MT2A* relative gene expression. (**H**) MT1/2 relative protein expression. (**I**) *FPN1* relative gene expression. Confluent BeWo b30 cells were incubated with MnCl_2_ and/or FeCl_2_ for 24 h. Relative gene expression was determined using RT-qPCR and normalized to *ACTB* (β-actin) as the housekeeping gene. Protein quantification via Western Blot was realized after β-actin normalization of determined protein levels. Shown is the mean + SD of at least three biological replicates. Statistical analysis was performed via unpaired *t* test with Welch’s correction and indicated as followed: as * *p* < 0.05, ** *p* < 0.01, and *** *p* < 0.005 compared to untreated control, §: compared to single Fe treatment.

mRNA as well as protein expression was determined in BeWo b30 cells after 24 h of TE exposure to specify which Mn and Fe transporters may be involved in single placental Mn and Fe transfer and in case of combined exposure (Figure 5). Considered transporters in this study were TfR, DMT1, ZIP14, and FPN and the transport and storage proteins MT isoforms 1A and 2A (MT1A, MT2A), FTH, and FTL. These are all discussed to be involved in Mn as well as Fe transfer and expressed in placental tissue [30,31,32,33]. For reasons of clarity and comprehensibility, *FTH*, *FTL*, and *ZIP14* gene expression is shown in the Supplementary Materials (Figure S2) since they were not regulated on the transcriptional level by Mn and/or Fe treatment in this study, as well as *MT1A* gene expression, which showed the same trend as *MT2A*.

Relative gene expression of *DMT1* and *FPN1* as well as FTL protein expression was significantly downregulated by 100 µM MnCl_2_ exposure of BeWo b30 cells for 24 h (Figure 5C,F,I). All other transporter-associated genes and proteins were not affected by MnCl_2_ incubation. FeCl_2_ exposure of BeWo b30 cells resulted in a significant downregulation of TfR on a transcriptional (100 µM FeCl_2_) and translational level (10 µM and 100 µM FeCl_2_) (Figure 5A,B) and *DMT1* gene expression was also downregulated after incubation with 100 µM FeCl_2_ (Figure 5C). Relative mRNA expression of *MT2A* was significantly upregulated by 100 µM FeCl_2_ (Figure 5G) and *FPN1* expression was significantly downregulated after 10 and 100 µM FeCl_2_ exposure (Figure 5I). As opposed to relative gene expression levels, translational regulation of FTH and FTL was strongly affected by Fe treatment. FTH and FTL protein expression was significantly increased 24 h after treatment with 10 µM or 100 µM FeCl_2_. Interestingly, the combination of 10 µM FeCl_2_ or 100 µM FeCl_2_ with 100 µM MnCl_2_ reduced FTH and FTL protein levels compared to single FeCl_2_ treatment (Figure 5E,F). Concurrent incubation of MnCl_2_ and FeCl_2_ also resulted in a significant downregulation of TfR on mRNA level (100 µM FeCl_2_ + 100 µM MnCl_2_) and protein level (10 µM FeCl_2_ + 100 µM MnCl_2_) as well as in *DMT1* mRNA expression (100 µM FeCl_2_ + 100 µM MnCl_2_) (Figure 5A–C). DMT1 protein expression showed a similar trend of downregulation for exposures to the combinations of both TEs (10 or 100 µM FeCl_2_ + 100 µM MnCl_2_) (Figure 5D). Additionally, protein expression of MT1/2 (Figure 5H) was significantly reduced by exposure to 100 µM FeCl_2_ + 100 µM MnCl_2_ while *MT2A* gene expression for 100 µM FeCl_2_ + 100 µM MnCl_2_ was significantly less affected compared to single Fe treatment (Figure 5G). *FPN1* mRNA expression (Figure 5I) was altered by 100 µM FeCl_2_ + 100 µM MnCl_2_, showing a significant downregulation compared to untreated cells. Compared to single FeCl_2_ treatment, TfR protein expression was significantly less affected by incubating 10 µM FeCl_2_ + 100 µM MnCl_2_ (Figure 5B). The same effect was observed in *MT2A* gene expression, where the combination of 100 µM FeCl_2_ + 100 µM MnCl_2_ resulted in a significantly lower upregulation compared to single FeCl_2_ treatment (Figure 5G). 

## 3. Discussion

While Mn is known to be essential for fetal development, concerns are rising about consequences for the developing fetus in the case of Mn overexposure in utero. Although it is known that Mn is able to cross the placenta and excess intrauterine Mn may lead to adverse pregnancy outcomes, it is surprising that underlying mechanisms of Mn transfer in placental barrier building cells have not been established yet. This key gap was addressed in this study using human BeWo b30 cells. BeWo b30 cells cultivated on microporous inserts are widely used as a model for the placental barrier because they form a confluent polarized monolayer resembling the structure and function of trophoblast cells in vivo [27,34]. Additionally, the relative transfer rates of small substances determined in the BeWo b30 monolayer correlate well with transfer indices from ex vivo placenta perfusions. BeWo cells further exhibit transcellular transport processes which are involved in placental nutrient transfer [35,36,37]. While data regarding Mn transfer across the placental barrier are rare, Mn translocation across the blood-brain, the blood cerebrospinal fluid (blood-CSF), or the intestinal barrier has already been elucidated in several studies [38,39]. It appears that multiple transfer pathways are involved, including facilitated diffusion and active transfer processes. The intestinal but also blood-brain barrier studies highlight an involvement of DMT1 and TfR in metal import and FPN for metal export [39,40]. Additionally, they have shown a competition for metal binding sites in metal transporters with Fe [38,39,41]. Therefore, we hypothesized that these transport pathways as well as competitive uptake between Mn and Fe would also occur in placental tissue. Fe transfer kinetics across the placental barrier and placental Fe homeostasis is already clarified in more detail in literature [30,31,32].

Fe is transported through TfR-mediated endocytosis, which is only unidirectional from mother to child. Once in the endosome, Fe is released from the Tf-Fe complex and subsequently transported into the cytosol from DMT1. If not stored in FTH or FTL, it can be transported to the fetus through FPN [22]. For placental Mn transfer, active transport mechanisms have been suggested mainly based on extrapolations from other TE transport or from rodent data [42], but a distinct proof of active transport and the involved transporters in humans is still lacking. Therefore, the comparison of Mn and Fe transfer and transfer interactions further help to clarify Mn transfer across the human villous trophoblast layer. 

Firstly, data presented in this work showed that the barrier integrity of the BeWo b30 cell layer was not perturbed by MnCl_2_ and FeCl_2_ treatment for up to 1000 µM since TEER values as well as capacitance values were not affected by TE treatment. In contrast, Bornhorst et al. showed that barrier building cells of the blood-CSF barrier, for example, are more susceptible to MnCl_2_ treatment, with 200 µM MnCl_2_ being sufficient to cause barrier leakage [38]. Similarly, incubation of differentiated Caco-2 with 15–50 µM Fe(II)/ascorbate led to disruption of the barrier after 2 h [43]. Barrier leakage or complete disruption was not reached in BeWo b30 cells with either MnCl_2_ or FeCl_2_ treatment, which hints that BeWo b30 trophoblasts may have adapted to relatively high levels of metal exposures. Since the placenta possesses lots of mitochondria, ROS accumulation is a constant condition which increases during gestation. Therefore, the placenta is provided with a high antioxidant capacity which allows efficient retention of metal-induced ROS [44,45]. The ability of BeWo b30 cells to survive high metal yields facilitates screening the transfer across a wide concentration range to show possible alterations in case cells are overexposed and to reveal a potential risk of an impaired fetal development because of high metal amounts reaching the fetal circulation. However, it has to be considered that concentrations from 100 µM MnCl_2_ applied in this study are not obtained under physiological relevant conditions in maternal serum or umbilical cord blood but in placental tissue with Mn amounts ranging from 62 ng/g to 89 ng/g dry weight [46,47,48,49,50]. 

In food but also in food supplements, the most important oxidation state of Mn is the divalent form. According to the Directive 2002/46/EC of the European Parliament relating to food supplements, MnCl_2_ is one of the used species for supplementation [51]. Since pregnant women are mainly exposed to divalent Mn through nutrition and drinking water, Fe was also applied in its divalent form in order to compare transfer mechanisms.

Transfer experiments conducted in this study pointed out distinct crossover kinetics for Mn or Fe, respectively (Figure 1). Expressing the data as percent of the applied dose revealed a constant Mn transfer that was independent of the applied concentrations but showed a time dependency with a higher transfer for longer exposure duration. A previous study on Mn transfer conducted in a perfused human placental lobule observed a restricted Mn transfer presumably following an active transport mechanism [15]. This mechanism was also proposed for other physiological barriers like the blood-brain or blood-CSF barrier [38,52]. However, Bornhorst et al., concluded that DMT1 is not the only involved transporter for Mn transfer at the blood-CSF barrier since incubation with a DMT1 inhibitor did not result in a restricted transfer [38]. In addition, previous rodent data or measurement of maternal serum or cord blood further corroborated the hypothesis of an active transfer of Mn across biological barriers [23,42,53]. However, for the placental trophoblast barrier, our data did not support a transport exclusively based on active pathways since no transfer restriction or concentration-dependency could be observed. Consequently, transcellular or paracellular transport mechanisms involving passive diffusion or transfer across the tight junctions should be considered as well in this context [36]. For first indications of transporter-mediated Mn transfer, we also incubated inhibitors for DMT1 and TfR (Ferristatin II) and FPN (Hepcidin) [54,55,56] in combination with Mn. Mn transfer and Mn bioavailability were not affected by inhibitor treatment (Figure S3) and in general, mRNA and protein expression of *TfR1*, *DMT1*, and *FPN1* was only slightly altered by Mn treatment. This is also underlining the hypothesis that Mn transfer is not only transporter mediated but likely involves a combination of active, transcellular, and paracellular transfer mechanisms, which needs to be further elucidated in future studies. 

This can also be confirmed focusing on Mn bioavailability. Cellular Mn concentration does not increase over time, indicating that Mn homeostasis is maintained through effective import and export mechanisms within the cells. However, while the BeWo b30 trophoblast transfer model is recapitulating the most relevant barrier layer for nutrient transfer to the fetus, further studies should be performed using a co-culture model of trophoblasts and endothelial cells to understand the contribution of the endothelial barrier to maternal-fetal Mn transfer [15,27,57].

Interestingly, different from Mn transfer, Fe transport was highly concentration-dependent until reaching a plateau at 100 µM FeCl_2_. The 100 µM FeCl_2_ might be a critical concentration above which the Fe transfer is more tightly regulated to maintain Fe homeostasis and avoid toxicity from excess metal exposure. Additionally, cellular Fe uptake was affected by inhibitor treatment, leading to a significant decreased Fe uptake after inhibition of TfR1 and DMT1 by Ferristatin II and also a trend for a slight increase in cellular Fe in combination of 100 µM FeCl_2_ with hepcidin, which is involved in FPN regulation (Figure S3) [56]. This is well in line with previous work showing that Fe transfer is a tightly regulated transporter-mediated process in many cells and tissues [58,59]. Heaton et al. proposed a passive diffusion for non-transferrin-bound Fe in BeWo b30 cells. Since passive diffusion is limited by molecular size, only unbound Fe can be transferred. It is therefore likely that in our study, Fe was also bound to Tf present in the cell culture medium to restrict the transfer across the BeWo b30 layer at high FeCl_2_ exposure concentrations, but this has to be verified by further studies focusing on Fe speciation [28,60]. 

Instead of focusing on isolated TEs, the more realistic exposure scenario for the population is the consumption of mixtures of TEs in the normal diet where Mn occurs mainly in cereal-based products or nuts, which are also rich in Fe [14,61]. This underlines the necessity to consider the entirety of all dietary ingredients. Additionally, Mn and Fe are typically added as micronutrients to food and supplements. Especially food supplements are taken in pregnancy in order to preserve a healthy future for the fetus and the pregnant woman [62] and it has also been postulated that dietary Mn absorption rate is also affected by Fe [63]. Therefore, combinations of different TEs and their effect on BeWo b30 trophoblasts were elucidated in this study. 

Concurrent Mn and Fe exposure of BeWo b30 cells showed that Mn and Fe transfer is influenced by the respective other TE. 10 µM FeCl_2_ significantly decreased transfer of 100 µM MnCl_2_ while 100 µM MnCl_2_ significantly increased Fe transfer (50 µM and 100 µM FeCl_2_). To understand if shared use of transporters is one possible mechanism of transfer interaction, we determined mRNA and protein expression of different transporters, transport proteins, and storage-associated proteins involved in TE transfer and homeostasis. In general, incubation of either Mn or Fe or the combination of both affected mRNA and protein expression only marginally except for protein expression of FTH and FTL. TfR protein expression was significantly downregulated by all tested single Fe conditions and adding 10 µM FeCl_2_ to 100 µM MnCl_2_ led to a decreased TfR downregulation compared to the single TE treatments. The same trend was also seen for *DMT1* mRNA expression, which was decreased in all conditions except 500 µM MnCl_2_ and the combination of 10 µM FeCl_2_ + 100 µM MnCl_2_. Together, these results indicate that BeWo cells responded to the increased Fe and Mn exposure in order to prevent intracellular accumulation of excess TE. However, quantifying the cellular amount, the cellular Fe concentration following 100 µM FeCl_2_ was significantly increased in combination with 100 µM MnCl_2_ (Figure 4). This observation can be explained by the decreased *FPN1* gene expression leading to a decreased Fe export. However, downregulation of *FPN1* gene expression was not distinguishable from single Fe treatment. It could be assumed that translational FPN1 regulation plays a bigger role, but data conducted in this study do not allow a clear explanation of the underlying mechanisms yet, since mRNA as well as protein expression of importers were not altered by concurrent Mn and Fe treatment compared to single Fe treatment as well. The cellular Mn content was slightly decreased in combination with 10 and 50 µM FeCl_2_, which indicates that Mn uptake is slightly compromised in combination with Fe. This is consistent with mRNA and protein expression data, which showed TfR1 and DMT1 downregulation for the combination but also for certain single TE treatments. Therefore, Mn import was restricted in combination with low Fe concentrations. Crucial for this observation may be the lower binding affinity of Mn to Tf, which is not as high compared to the competing Fe present in the medium and therefore inhibiting Mn transfer by the TfR [64]. It is still unclear why this effect was not observed with higher Fe concentrations investigated in this study. 

Several studies have shown that in case of high metal exposure, importers like *DMT1* and *TfR1* are downregulated and the export by *FPN1* is upregulated on mRNA level in order to avoid metal accumulation [65,66]. However, in this study, mRNA expression of the exporter *FPN1* was downregulated by Fe treatment which is observed in other studies in case of Fe deficiency [67]. Li et al. also observed *FPN1* downregulation in BeWo cells after treatment with human holo-transferrin but the underlying mechanism cannot be explained yet [68]. One possible hypothesis may be that trophoblast cells are preventing an influx of excess metal to avoid oxidative stress, but simultaneously decrease export to store Fe, which is left in the cell to prevent Fe deficiency and maintain proper cell function. On the other hand, it could also be that the export is reduced to avoid overexposure of the sensitive developing fetus. *FPN1* downregulation was previously observed in studies from Sangkhae et al., which showed that under severe maternal Fe deficiency, the placenta downregulates Fe export to maintain Fe homeostasis and to ensure fetal development [69]. Since Fe transfer was enhanced in the presence of Mn while *FPN* mRNA expression was downregulated, it can be suggested that Fe transfer across the trophoblast layer is not exclusively mediated by Fe exporters but involves other mechanism like passive diffusion. 

Focusing on Fe treatment, FTH and FTL protein expression was strongly upregulated in both Fe conditions which additionally indicates that Fe is incorporated into FTH and FTL subunits to prevent Fe accumulation [70,71]. On the contrary, FTL protein expression was significantly decreased after treatment with 100 µM MnCl_2_. In comparison with single Fe treatment, FTH and FTL protein expression was less affected in combination with Mn. However, it is not obvious if Fe storage is decreased due to the presence of Mn or if Fe is released from FTH/FTL through lysosomal degradation [70]. Fe is stored in FTH or FTL by Fe binding to the iron regulatory protein (IRP), resulting in a release from the 5′ untranslated region (5′-UTR) from *FTH/FTL* mRNA [58]. Venkataramani et al. observed *FTH* downregulation in neuronal SH-SY5Y cells after Mn treatment, which is in line with the results in this study [72]. The authors concluded that the 5′-UTR of the *FTH* mRNA transcript was blocked by Mn, thereby inhibiting FTH protein expression [72]. Tai et al. also revealed that low Mn doses were sufficient to increase autophagic ferritin degradation and free Fe pool in SH-SY5Y cells, which in turn might lead to increased ROS formation [73,74,75]. This supports also the higher Fe bioavailability in the trophoblasts measured in this study. Interestingly, *MT2A* gene expression is highly induced after treatment with 100 µM FeCl_2_ but not in the presence of Mn. Since MTs are metal-binding proteins able to scavenge ROS, because they are rich in cysteine, it could be assumed that incubating two TEs would cause a higher induction because there is a higher potential for ROS formation [76]. However, there was no clear evidence why *MT2A* gene expression was less affected in the presence of Mn.

## 4. Conclusions and Outlook

**Figure 6:**
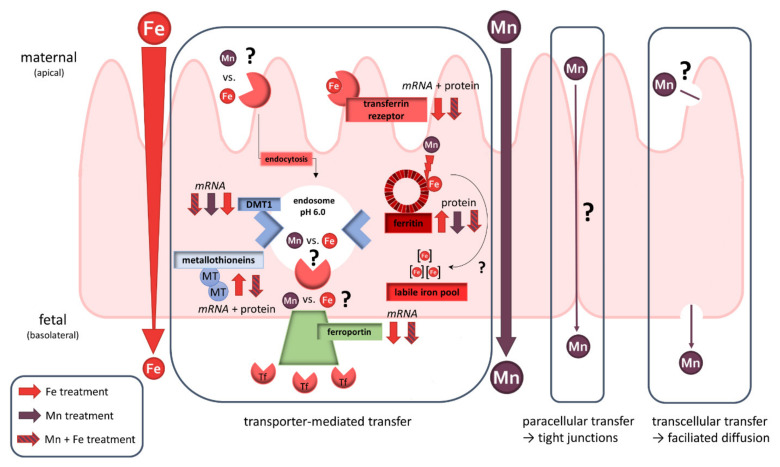
Schematic overview of placental transfer processes of Mn and/or Fe and their effect on transporter expression in the BeWo b30 trophoblast. While Fe seems to be transferred transporter-mediated, Mn transfer appears to follow several transfer mechanisms like paracellular, transcellular, and transporter-mediated pathways.

Although many studies already discussed Mn and Fe transfer across physiological barriers, this study is to our knowledge the first to investigate Mn transfer in comparison to the already clarified Fe transfer in human trophoblasts. Since interaction of these TEs in regard of shared transport systems has been elucidated in different tissues and cell types before, this study highlighted the role of Mn and Fe transfer interactions in BeWo b30 cells as well. By applying the widely used BeWo b30 Transwell^®^ model, we were able to show that Mn transfer differs from Fe since it is not a restricted, concentration-dependent mechanism. Analysis of mRNA as well as protein expression of discussed transporters and storage proteins did not reveal one single mechanism that contributes to Mn transfer across the trophoblast. However, we found that placental Mn transfer involves a combination of transcellular, paracellular, and active transport mechanisms including metal transporters (Figure 6). Since we focused on active transfer mechanisms in BeWo b30 cells, transcellular and paracellular processes should be the focus of future transfer studies as well as Mn and Fe speciation analysis and further mechanistic studies on adverse effects. Additionally, we could show that Mn and Fe interactions are also taking place in trophoblast cells, which is a crucial observation highlighting that it is important to consider not only one single TE. This can also be underlined by our single TE transfer studies which put an emphasis on the fact that not every TE is transferred the same way and extrapolation of mechanisms from single TE on other TEs should be avoided. Since placental Mn transfer data are scarce, putting Fe transfer in relation was necessary to get ideas for possible mechanisms and also revealed important interactional processes. TE interactions in pregnancy should be a major part of future research studies to understand homeostatic alterations caused by TE mixtures in order to improve the assessment of micronutrient requirements during pregnancy. 

## 5. Materials and Methods

### 5.1. Cultivation of BeWo b30 Cells

The human placental choriocarcinoma cell line BeWo subclone b30 was cultivated as described previously [27]. Briefly BeWo b30 cells were cultured using Ham’s F-12K medium (Thermo Fisher Scientific (Gibco), Schwerte, Germany) supplemented with 1% fetal calf serum (FCS; Biochrom GmbH, Berlin, Germany), 1% penicillin/ streptomycin and 2 mM L-glutamine (Sigma Aldrich, Steinheim, Germany). Cells were sub-cultured twice a week using 0.05% trypsin-EDTA solution (Sigma Aldrich, Steinheim, Germany) with a medium change every two days and cultured in a humidified incubator at 37 °C with 5% CO_2_. 

### 5.2. Cytotoxicity Testing for Dosage Regimen

Stock solutions of MnCl_2_ (MnCl_2_·4H_2_O, 99.9% trace element basis, Honeywell^TM^, Morristown, NJ, USA) and FeCl_2_ (FeCl_2_·4H_2_O, 99.9% trace element basis, Merck Millipore, Darmstadt, Germany) were prepared freshly before the experiment using sterile purified water (18 MΩ). The abbreviations Mn and Fe used in this study refer to both metals in the divalent form (Mn(II) and Fe(II)). For cytotoxicity testing via Hoechst Assay, 31,000 cells/cm^2^ were seeded in 96-well plates in order to reach confluency after three days [27]. One hour after medium change, cells were incubated with different concentrations of MnCl_2_ and FeCl_2_ ranging from 100–1000 µM for up to 24 h. In short, Hoechst assay was carried out by fixing cells using 4% formaldehyde followed by a permeabilization step with Triton™ X-100 (Sigma Aldrich, Steinheim, Germany) to allow Hoechst staining (Bisbenzimide H33258, Calbiochem, Sigma Aldrich, Steinheim, Germany) to interact with the DNA of living cells [77]. 

### 5.3. Mn and Fe Transfer across the BeWo b30 Cell Layer

For transfer experiments, 8.5 × 10^4^ cells were seeded in endothelial growth medium MV (EC) supplemented with 1 vial SupplementMix according to the manufacture’s manual (PromoCell, Heidelberg, Germany) and 1% penicillin/streptomycin on the apical side of microporous inserts (Transwells^®^ with a polycarbonate membrane, 0.4 µm pore size, 1.12 cm^2^ growth area, Corning Life Sciences, Amsterdam, The Netherlands). Before cell seeding, inserts were coated using a 50 µg/mL solution of human placental collagen for 1 h at 37 °C (Sigma Aldrich, Steinheim, Germany). Three days after seeding, the medium was changed 2 h before TE treatment. Barrier tightness was monitored right before and during an experiment by measuring transepithelial electrical resistance (TEER) using the cellZscope^®^ device (nanoAnalytics, Münster, Germany). Additionally, barrier integrity was proven by sodium fluorescein translocation and staining of the tight junction and microtubule proteins γ-catenin and tubulin as described elsewhere [27]. For transfer studies, cells were treated with non-cytotoxic concentrations of MnCl_2_ (100–1000 µM) and FeCl_2_ (10–500 µM) on the apical side. Samples were taken from the apical and basolateral compartment 6, 24, and 48 h after treatment. Permeability coefficients were calculated as followed:

(1) $$P=\frac{\Delta Q\;\left[µg\right]}{A\;\left[{\mathrm{cm}}^{2}\right]*\;c_{0}\left[\frac{µg}{{\mathrm{cm}}^{-3}}\right]*\Delta t\;\left[s\right]}$$

with the permeability (*P*; cm s^−1^) determined as the quotient of the basolateral amount (Δ*Q*; µg; normalized to sample volume) and the product of the area of the microporous insert (*A*; cm^2^), the apical concentration at the start of the incubation (*c*_0_; µg cm^−3^) and the respective time point (*t*; s)

To quantify the TE content in cells grown on inserts, the membrane was cut out with a scalpel and washed in ice-cold PBS to remove remaining medium. Cells were stored at −20 °C until lysis. For cell lysis, cells were digested using a lysis buffer consisting of 1 mM TRIS (Roth, Karlsruhe, Germany), 0.1 M NaCl (Roth, Karlsruhe, Germany), 1 mM EDTA disodium salt (VWR, Darmstadt, Germany), and 0.1% Triton™ X-100. Lysates diluted in 2% HNO_3_ were measured using an inductively coupled plasma-optical emission spectrometer (ICP-OES; Spectro, Krefeld, Germany) or Agilent ICP-MS/MS Triple Quad system (ICP-QQQ-MS 8800, Agilent, Waldbronn, Germany). Measurement parameters can be found in the Supplementary Materials (Tables S3 and S4). TE amounts were validated by measuring acid-assisted digested certified reference material BCR^®^-274 (Single Cell Protein, Institute for Reference Materials and Measurement of the European Comission, Geel, Belgium) and SRM^®^-1640a (Trace Elements in Natural Water, National Institute of Standards & Technology, Gaithersburg, MD, USA). The cellular TE concentration was normalized to the protein amount determined by BCA-assay. 

### 5.4. Transporter Inhibition Study

Transporter inhibition was determined according to single or combined TE incubation as described in 5.3. Furthermore, Hepcidin/LEAP-1 (Human) (Axxora-Enzo Life Sciences, Lörrach, Germany) was incubated 1 h before and Ferristatin II (NSC 8679, NCI Developmental Therapeutic Program) simultaneously in combination with MnCl_2_ or FeCl_2_ on the apical side of the microporous insert. After 6 and 24 h, samples were taken from the apical as well as basolateral compartment and quantified using ICP-OES. Cellular Mn and Fe uptake was also assessed after cell lysis. 

### 5.5. Quantitative Real-Time PCR Analysis

Expression of human metal transport and storage-associated genes was investigated from TE-exposed cells cultivated on microporous insert. Total RNA was isolated using NucleoSpin^®^ extraction kit (Marcherey-Nagel GmbH & Co. KG, Düren, Germany) and RNA yield was determined with Nano Drop One Spectrometer (Thermo Fisher Scientific, Waltham, MA, USA). RNA with absorption ratios A260/A280 and A260/A230 between 1.8–2.2 were considered as pure. For cDNA synthesis, 1 µg RNA was transcribed with a High-Capacity cDNA Reverse Transcription Kit (Applied Biosystems^TM^, Thermo Fischer Scientific, Waltham, MA, USA) according to the manufacturer’s protocol. Prior to RT-qPCR analysis with iQ^TM^ SYBR^®^ Green Supermix (Bio-Rad Laboratories Inc., Hercules CA, USA) as fluorescence probe, primer efficiency was validated by a cDNA concentration curve and primer concentrations ranging from 0.2–0.6 µM and product purity was verified by gel electrophoresis. Only primers with an efficiency between 90–120% were used (Table S5). The applied temperature program carried out on the AriaMx Real-Time PCR System (Agilent, Waldbronn, Germany) included a polymerase activation step at 95 °C for 3 min, DNA denaturation at 95 °C for 30 s, primer annealing at 56 °C for 1 min, and extension phase at 72 °C for 15 s. The cycle from DNA denaturation to extension was repeated 37 times. After each extension phase, fluorescence intensity was measured. For melting curve analysis, DNA was denaturized at 95 °C for 1 min with a subsequent increment from 60–95 °C within a minute. C_q_ values determined were normalized to β-actin as the housekeeping gene and evaluated in consideration of the primer efficiency.

### 5.6. Western Blot Analysis

For Western Blot analysis cell pellets, pelletized from culture dishes, were lysed in ice-cold RIPA buffer in combination with sonification using an ultrasonic probe (6 s, amplitude: 100%, cycle: 0.5). Protein amounts from 10–30 µg denaturated with 5x Laemmli buffer (12.5% β-mercaptoethanol (*v*/*v*), 10% SDS (*w*/*v*), 50% Glycerol (*v*/*v*), 0.2 M Tris (pH 6.8), 0.625% Bromphenol blue) were applied for SDS-PAGE. Protein transfer on a nitrocellulose blotting membrane (0.2 µm pore size, Amersham™ Protran™, Merck, Darmstadt, Germany) via tank blotting (Bio-Rad Laboratories Inc., Hercules, CA, USA) was verified using 0.2% Ponceau staining. After blocking membranes in 3% (*w*/*v*), non-fat dry milk in 1x Tris buffered saline containing 0.1% (*v*/*v*) Tween^®^ 20 (T-TBS) for 1 h at RT, primary antibodies diluted in blocking solution were incubated at 4 °C over night. Recombinant anti-β-actin (1:2500, ab115777, Abcam, Cambridge, UK), anti-Metallothionein (1:500, UC1Mt, Invitrogen, Thermo Fischer Scientific, Waltham, MA, USA), anti-DMT1 (1:500, ab55735, Abcam, Cambridge, UK), anti-TFRC (1:1000, D7G9X, Cell Signalling, Danvers, MA, USA), anti-FTH1 (1:500, D1D4, Cell Signalling), and anti-FTL (1:500, ab69090, Abcam, Cambridge, UK) were used as primary antibodies. Horseradish peroxidase (HRP)-conjugated goat anti-mouse or goat anti-rabbit antibodies (1:10000, Bio-Rad Laboratories Inc., Hercules, CA, USA) were incubated as secondary antibodies for 1 h at RT. Since the primary antibodies against TfR, FTH, and FTL are very potent and belong to the same species as the first antibody against β-actin, the secondary HRP goat anti-rabbit antibody was diluted 1:2500. Chemiluminescence detected with Amersham Imager 600 (GE Healthcare, Chicago, IL, USA) was achieved by incubation of immunoblots with Clarity™ Western ECL Substrate (Bio Rad Laboratories Inc., Hercules, CA, USA). Protein bands were quantified using ImageJ software and normalized to β-actin as loading control. 

### 5.7. Statistical Analysis

Statistical analysis was performed using GraphPad Prism 9 Software (GraphPad Software, La Jolla, CA, USA). Unless otherwise stated, data are shown as mean ± SD and significance values are depicted as * *p* < 0.05, ** *p* < 0.01, and *** *p* < 0.005 compared to untreated control.

## Data Availability

Not applicable.

## Supplementary Materials

The following supporting information can be downloaded at: https://www.mdpi.com/article/10.3390/ijms23063296/s1.
