# Supplementary material for: Differences and Interactions in Placental Manganese and Iron Transfer across an In Vitro Model of Human Villous Trophoblasts
Source: Int J Mol Sci. 2022 Mar 18;23(6):3296. doi: 10.3390/ijms23063296 (PMC8951728; doi:10.3390/ijms23063296)
Supplement: Supplementary file 1 [file ijms-23-03296-s001.zip › ijms-1605686-supplementary.pdf]

Supplementary data

International Journal of Molecular Sciences – Special Issue: Metals and Cellular Redox and Immunity Status

Differences and interactions in placental manganese and iron transfer across an *in vitro* model of human villous trophoblasts

Vivien Michaelis[1], Leonie Aengenheister[2,3], Max Tuchtenhagen[4,5], Jörg Rinklebe[6], Franziska Ebert[5], Tanja Schwerdtle[4,5,7], Tina Buerki-Thurnherr[2] and Julia Bornhorst[1,4,*]

[1] Food Chemistry, Faculty of Mathematics and Natural Sciences, University of Wuppertal, Gaußstraße 20, 42119 Wuppertal

[2] Empa Swiss Federal Laboratories for Materials Science and Technology, Particles-Biology Interactions, Lerchenfeldstraße 5, 9014 St. Gallen, Switzerland

[3] Luxembourg Institute of Health, Rue Thomas Edison 1 A-B, 1445 Strassen, Luxembourg

[4] TraceAge-DFG Research Unit on Interactions of Essential Trace Elements in Healthy and Diseased Elderly (FOR 2558), Berlin-Potsdam-Jena-Wuppertal, 14558 Nuthetal, Germany

[5] Department of Food Chemistry, Institute of Nutritional Science, University of Potsdam, Arthur-Scheunert-Allee 114-116; 14558 Nuthetal, Germany

[6] Laboratory of Soil-, and Groundwater-Management, School of Architecture and Civil Engineering, Institute of Foundation Engineering, Water- and Waste-Management, University of Wuppertal, Pauluskirchstraße 7, 42285 Wuppertal, Germany

[7] German Federal Institute for Risk Assessment (BfR), Max-Dohrn-Straße 8-10, 10589 Berlin, Germany

*Correspondance: bornhorst@uni-wuppertal.de

Results

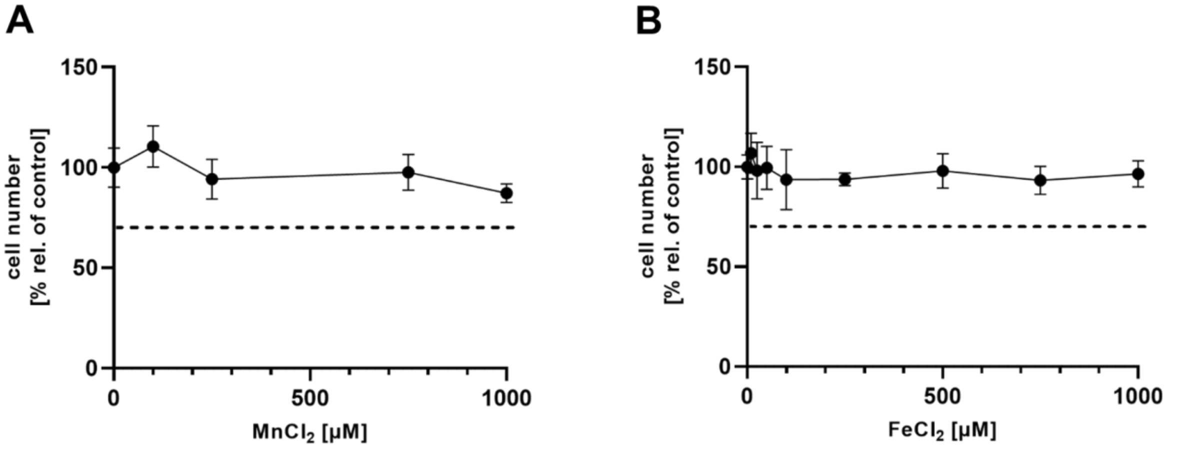

Figure S1: $MnCl_2$ (A) and $FeCl_2$ (B) cytotoxicity in confluent BeWo b30 cells after 24 h assessed using Hoechst Assay. Shown is the mean ± SD of three independent experiments.

Table S1: Bioavailability of $MnCl_2$ after 24 and 48 h in BeWo b30 cells. Data shows the mean ± SD of at least three independent experiments with two replicates each.

**Incubated $MnCl_2$ [µM]**	**Mn bioavailability [pg/ µg protein]**	
	**24 h**	**48 h**
0	29.2 ± 9.5	63.4 ± 6.1
100	121.0 ± 33.7	143.7 ± 48.9
500	-	343.7 ± 50.9
750	-	541.2 ± 130.8
1000	-	705.0 ± 280.4

Table S2: Bioavailability of $FeCl_2$ after 48 h in BeWo b30 cells. Data shows the mean ± SD of at least three independent experiments with two replicates each.

**Incubated $FeCl_2$ [µM]**	**Fe bioavailability [pg/ µg protein]**
	**48 h**
0	64.1 ± 12.0
10	82.5 ± 23.4
50	185.1 ± 67.2
100	290.4 ± 29.4
500	1092.6 ± 180.6

Table S3: ICP-OES parameters.

**Parameter**	**Working conditions**
Plasma power [W]	1400
Cooling gas flow [L/min]	12.00
Auxiliary gas flow [L/min]	1.00
Nebulizer flow [L/min]	1.00
Nebulizer type	MicroMist®
Torch alignment	Axial<br>Basic settings: 0.2 mm horizontal<br>3.8 mm vertical<br>3.0 mm distance
Element wavelengths	Mn: 257.611 / 259.373<br>Fe: 259.940 / 238.204<br>Cu: 324.754 / 224.700<br>Zn: 202.548 / 213.856

Table S4: ICP-MS/MS parameters.

Parameter	Working conditions
Plasma power [W]	1550
Cooling gas flow [L/min]	15.00
Auxiliary gas flow [L/min]	0.90
Nebulizer flow [L/min]	1.00
Nebulizer type	MicroMist®
Spray chamber	Scott-type
Spray chamber temperature [°C]	2
Makeup Gas flow [L/min]	0.26
Torch alignment	Axial
Gas mode	He (CRC-mode)
He gas flow [mL/min]	3
Transitions	Mn: 55 → 55<br>Fe: 56 → 56<br>Cu: 63 → 63<br>Zn: 66 → 66<br>Internal Standard (IS)<br>Ge: 72 → 72<br>Rh: 103 → 103

Table S5: Primer sequences of human metal transport and storage-associated genes.

Gene name	Association	Primer sequences (5′ → 3′)	Amplicon length (bp)	Efficiency [%]
ACTB	β-actin	Forw.: CATCCGCAAAGACCTGTACG<br>Rev.: TCTCCTTCTGCATCCTGTCG	86	103.4
DMT1	divalent metal transporter 1	Forw.: AGTTGGCTATCATCGGCTCA<br>Rev.: TCTGCAATGGTGATGAGAACG	115	106.8
TfR1	transferrin receptor 1	Forw.: TGAGAGGTACAACAGCCAACT<br>Rev.: CACGAGCAGAATACAGCCAC	107	108.1
SLC40A1 (FPN1)	ferroportin 1	Forw.: TCGCCTAGTGTCATGACCAG<br>Rev.: TTGCAGAGGTCAGGTAGTCG	85	104.6
SLC39A14 (ZIP14)	zrt-, irt-related protein 14	Forw.: CAGTCACCATGAAGCTGCTG<br>Rev.: GGTTCTCCATAAGCCAAGCAG	80	113.3
MT1A	metallothionein 1A	Forw.: GCAAAGGGGCATCAGAGAAG<br>Rev.: TGGGTCAGGGTTGTATGGAA	119	118.9
MT2A	metallothionein 2A	Forw.: GTTGCCTCCTCAGTGATCCT<br>Rev.: GGCGGCAGAGATGAGTACTA	77	105.3
FTH1	ferritin heavy chain 1	Forw.: CATCAACCGCCAGATCAACC<br>Rev.: CACATCATCGCGGTCAAAGT	82	111.7
FTL	ferritin light chain	Forw.: ATCTTCTCGGCCATCTCCTG<br>Rev.: TGGTTGGCAAGAAGGAGCTA	70	103.2

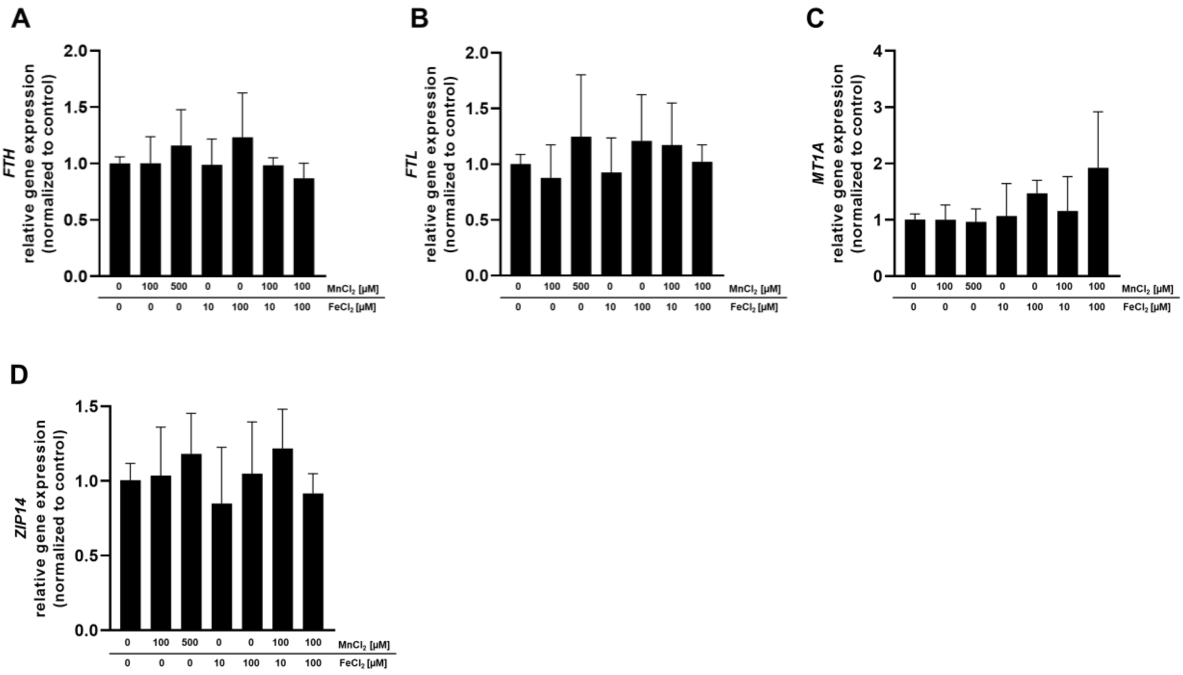

Figure S2: Relative mRNA levels of (A) *FTH*, (B) *FTL, (C) MT1A, (D) ZIP14*. Confluent BeWo b30 cells were incubated with $MnCl_2$ and/ or $FeCl_2$ for 24 h. Relative gene expression was determined using RT-qPCR and normalized to *ACTB* (β-actin) as housekeeping gene. Shown is the mean + SD of at least three biological replicates.

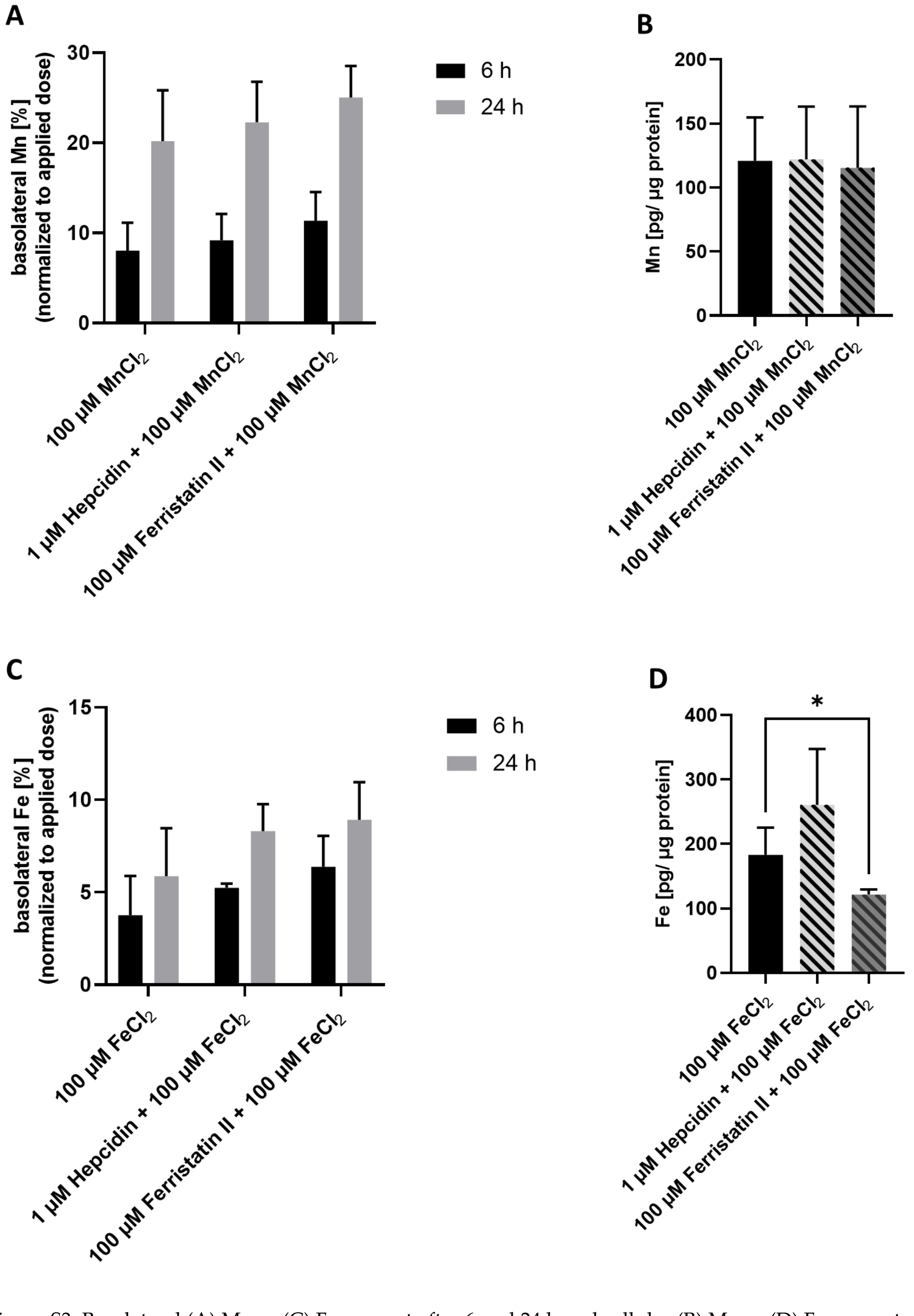

Figure S3: Basolateral (A) Mn or (C) Fe amount after 6 and 24 h and cellular (B) Mn or (D) Fe amount after 24 h of BeWo b30 cells incubated with inhibitors for DMT1 and TfR (Ferristatin II) and FPN (Hepcidin) in combination with $MnCl_2$ or $FeCl_2$. Shown is the mean + SD of at least two independent experiments with two biological replicates each. Statistical analysis based on an unpaired t test with Welch's correction is depicted as followed: *: compared to untreated control.
